# Opportunities and Challenges of Generative AI in Postgraduate Health Professions Education Assessments From Educator and Learner Perspectives: Qualitative Study

**DOI:** 10.2196/87121

**Published:** 2026-05-06

**Authors:** Carys Phillips, David Harrison

**Affiliations:** 1 Research Department of Medical Education, University College London, Education RCP, 11 St Andrews Place, London, WC1E 6BT, United Kingdom, 44 7713003204

**Keywords:** assessments, AI, artificial intelligence, GenAI, generative artificial intelligence, postgraduate, health professions education, medical education, written assessment, essay

## Abstract

**Background:**

The application of artificial intelligence (AI) is increasingly valuable as a tool and assistant in many areas of clinical and academic medicine. Generative AI (GenAI) creates new content used by large language models, which can generate language that strongly resembles or even improves on that of humans. Learners and educators in many areas of education are using GenAI for essays and assessments, raising issues regarding learning and assessment. GenAI is also raising new concerns in health professions education (HPE), an area of health professions training that sometimes has different aims and assessment methods compared to its clinical counterparts. HPE needs to assess levels of knowledge and understanding of pedagogy, and the use of GenAI presents challenges to its current assessments, which are predominantly written.

**Objective:**

The study aimed to investigate educators’ and learners’ perspectives on the opportunities and challenges presented by GenAI in postgraduate HPE assessments. It particularly focused on perspectives of how GenAI may influence the future of assessment and essay-based assessments in HPE.

**Methods:**

Informed by a constructivist paradigm, a qualitative approach was adopted, undertaking 8 semistructured interviews conducted via Microsoft Teams. Purposive sampling ensured a mixture of educators and learners in current HPE courses from a range of health care professions. Data were thematically analyzed.

**Results:**

There was no difference between educator and learner perspectives. Four themes were identified: AI is here, students are at a disservice if we do not embrace it; AI as an opportunity to rethink HPE assessments; AI is a “gray area”; and AI is fallible.

**Conclusions:**

The findings present AI as an external catalyst, highlighting the current internal desire for assessment change within HPE. It offers opportunities for creative, authentic assessments that reflect real-life academic and clinical practice, aiming to develop competent future HPE educators and keep courses relevant. These findings contribute to the debate around the future potential and development of AI in HPE assessments.

## Introduction

### Background

National Health Service England’s 2023 *Long Term Workforce Plan* outlines the need to develop an increasing number of skilled health care professionals to address the projected workforce shortfall from imminent “demographic pressures” and “changing burden of disease” [1]. Subsequently, education and training needs are predicted to increase between 50% and 65% by 2030 and 2031 across all health care professions [1], which necessitates a “high-quality educator workforce” [2]. More recently, the *10 Year Health Plan for England* highlighted plans to “modernize postgraduate medical education,” as well as work with educational institutes to “overhaul education and training curricula” for health care professions, including training in artificial intelligence (AI) use [3]. This increasing demand for modern educators means that health professions education (HPE) programs must be confident they are appropriately assessing whether learners achieve required professional education standards in the light of a constantly changing world.

AI plays a crucial role in this process, both by driving change and providing advanced tools and technologies that support personalized learning, efficient assessment, and continuous improvement in educational practices. More specifically, “generative AI” (GenAI) is now widely used and rapidly developing, capable of creating new content, including text, images, and other media. Within GenAI, natural language processing allows computers to understand human language; analyze, process, and interpret text; extract meaning; and perform tasks previously thought to require real intelligence [4]. Large language models (LLMs) are a further type of GenAI that perform natural language processing tasks by using “artificial intelligence algorithms to generate language that resembles that produced by humans” [4].

GenAI has significant ramifications within education, including HPE, which have become noticeable since the increased use following the release of the updated LLM by OpenAI in November 2022, ChatGPT-3.5. There has been subsequent development of LLMs internationally. Concerns arise regarding inequity for learners in barriers accessing GenAI, but these will likely diminish with their ongoing development, driving down costs and increasing accessibility. Crucially, the use of GenAI has triggered concerns among many HPE educators around how AI will influence teaching and learning and, especially, assessments. Current forms of assessment in HPE, especially at master’s level, are traditionally essay-based, incorporating reports, literature reviews, reflective pieces, and theses. By contrast, clinical qualifications tend to use “objective” examinations and work-based assessments. Hence, within HPE, there are increased concerns regarding potential academic misconduct in essay-type assessments if students use AI [5] and the implications for the quality and competence of these future educators when they enter the workforce.

### Cheating and AI

Concerns about cheating in assessments are not new. Essay-based assessments have always been open to “contract cheating,” when a student submits work written by someone else, but GenAI has made this more accessible [6] and has blurred the lines between cheating, plagiarism, and a “helping hand.” Among academics, vacillation is rife over whether GenAI is a “game-changer” and represents an end to the essay-based assessment or adds little to the multiple ways students can already cheat [7]. Besides potential academic misconduct, there are other considerations related to the use of AI within HPE assessment: privacy risks involving sensitive data [8], bias arising from the data on which the AI systems are trained [9], and “hallucinations” whereby AI creates false information and fabricates references [10].

“AI-output detectors” detect the use of AI in academic work, with varying results against humans in detecting AI-generated questions [11], but they have successfully discriminated between most original and AI-generated abstracts [12]. Indeed, there are platforms to help bypass AI-output detectors, so in this escalating arms race, the speed of AI’s sophisticated evolution makes it almost impossible for output detectors to stay ahead. This increases concerns about academic misconduct or misapplication in HPE assessments and questions whether educators or software will be able to detect its use.

### AI Literacy

AI literacy is “the ability to understand, use, monitor, and critically reflect on AI applications without necessarily being able to develop AI models themselves” [13] and is necessary for both learners and educators to effectively use AI within assessments. Although self-ratings for AI-knowledge in health professions students are low [14], learner perceptions toward AI are positive [15], wanting AI to be incorporated into medical school curricula [14,16,17]. If AI is formally incorporated into the curriculum, this will affect both *how* and *what* we assess, and educators will need to feel confident in AI literacy, as there are a multitude of ways to incorporate AI into assessment [18]. Tlili et al [19] identified the need to “upskill” educators on AI’s practicalities and how to design and teach it in curricula. Educator training would improve AI literacy and address faculty concerns related to using AI, such as misinformation or academic misconduct [20,21]. Current assessments within HPE are unlikely to be suitable to assess AI literacy, yet not incorporating AI in learning and assessment will likely impede future educators, learners, and patients, given AI’s growing presence.

### Communities of Practice

Communities of practice (CoP) are “groups of people who share a concern or a passion for something they do and learn how to do it better as they interact regularly” [22], and here it is used as a framework to consider how HPE forms CoP to co-construct knowledge and stay “ahead of the curve” in the face of the complex challenges, changes, and opportunities AI presents.

This research focuses on the views of opportunities and challenges within HPE assessment of GenAI (AI here refers to GenAI unless specified otherwise), specifically LLMs. The approach analyzes participants’ perspectives on the concept of LLMs in the context of HPE assessments. Discourse within the educator community is currently evolving and full of uncertainty, particularly in exploring how AI may specifically influence written HPE assessments, so we ask the question: what are educators’ and learners’ perspectives on the opportunities and challenges presented by GenAI in postgraduate HPE assessments?

## Methods

### Methodology

This research focused on exploring perspectives from a constructivist paradigm, using qualitative methodology. It aimed to explore educators’ and learners’ perceptions of how AI may influence HPE assessments, rather than testing knowledge of AI. Participants’ experiences are explored with the idea of effecting potential change—whether in an approach to future assessment or attitudes to AI within the HPE community. Methods are reported in line with the Consolidated Criteria for Reporting Qualitative Research (COREQ) checklist (Checklist 1) [23].

### Sampling and Recruitment

Educators currently teaching or assessing a postgraduate HPE course and current learners enrolled in a postgraduate HPE course were invited via purposive sampling to participate in semistructured interviews exploring educators’ and learners’ perspectives. Educators and students from different HPE courses across the United Kingdom were invited to avoid response bias specific to perspectives from a single institution. As GenAI was a relatively new concept at the time of interviews (January to March 2024), the inclusion criteria specified that only educators and postgraduate learners currently involved in a course were invited to participate. Exclusion criteria were any undergraduate students or individuals either teaching or being taught by CP. As there was no 1 overall group from which to recruit participants, purposive sampling ensured the invitation reached those who met the criteria. Participants were recruited through an email invitation sent by CP, and participants were invited to forward the invitation to colleagues to encourage snowball sampling. CP and administrators of appropriate HPE social media groups distributed the invitations through their respective health profession education social media channels (Microsoft Teams and WhatsApp) to widen the invitation’s reach. It was unclear how many were approached via this method, as there are multiple educators and learners on such channels.

### Research Team and Reflexivity

CP held roles as clinician, researcher, and educator. She was both an educator and learner on different postgraduate HPE courses. The research was for her master’s thesis (for MSc in medical education at University College London/Royal College of Physicians), with prior experience in qualitative research. DH was her supervisor and educationalist.

Specific ethical implications were considered, as recruitment methods included inviting some participants who were colleagues of the interviewer (CP). The participant information leaflet clearly outlined the intent of the research, and alongside the email invitation, it was made clear that the study was part of a master’s thesis and participation was voluntary. The invitation was also disseminated by administrators via wider social media channels to increase the uptake of participants, especially those not known to the interviewer.

Reflexively, CP was aware that their dual role as a current learner and as an educator with an interest in assessment and AI could potentially be intimidating in interviews if participants did not feel knowledgeable about AI, thereby creating a barrier to honesty. However, the dual role could also have been advantageous if interviewees felt that CP had an understanding of their role and, therefore, may have felt more comfortable.

DH was the supervisor for this research and an educationalist with assessment responsibilities and an interest in how AI is impacting assessment. DH was very aware of the potential for a conflict of interest—particularly if current assessment methods were perceived as no longer fit for purpose. DH had no knowledge or contact with the participants, and none of them were involved in the assessment process of any programs involving DH.

### Methods of Data Collection

Semistructured interviews were conducted by CP as the sole interviewer on Microsoft Teams and auto-transcribed. The transcripts were then pseudonymized and edited by the interviewer to ensure they were transcribed verbatim. Interviews were audio-video recorded with the camera off to encourage participants to speak freely and to increase confidentiality, and recordings were deleted at the end of the withdrawal period. Only the interviewer (CP) and the participant were present during the interview, and there were no repeat interviews. CP made research notes during the interviews to refer back to during analysis. The length of interviews can be found in the Results section. The interview schedule (Multimedia Appendix 1) was developed by CP to answer the research questions while allowing flexibility, with initial questions being reviewed and revised based on feedback from DH. The interview schedule started with more open-ended questions on the participants’ current roles and HPE assessments, before exploring their knowledge, perceptions, and use of AI both personally and within their communities. Subsequent questions were more structured to address the research questions and examined opportunities and challenges of AI within HPE assessments for both educators and learners. The interview schedule was not piloted due to concerns of low recruitment but was revised based on feedback from DH, and the semistructured nature of the interview allowed for sufficient flexibility. There was no member checking of transcripts, both for feasibility and to avoid participants reconsidering their original views.

### Data Analysis

Coding was undertaken by 1 data coder (CP) using NVivo 14 (Lumivero), as part of an independent master’s thesis. Data were analyzed using Braun and Clark’s thematic analysis [24], following the 6-phase process and using a predominantly inductive approach to reflect the relative novelty of the topic at the time.

In phase 1, CP immersed herself in the data by editing and rereading the transcripts. In phase 2, in keeping with Braun and Clarke’s [24] thematic analysis, “codes” were given to lengths of text relevant to the research question. Data that captured similar meanings were assigned to the same code. In phase 3, codes were then constructed into subthemes and then grouped under themes. There was no participant checking of codes. Associated codes from themes and subthemes helped identify illustrative quotes outlined in the Results section. In phase 4, themes were reviewed, and as CP was the only coder, there was no interresearcher triangulation of themes and, therefore, no disagreements. The limitations of having a single coder in triangulation and the potential for bias were acknowledged. However, data analysis started after a couple of interviews had been conducted and transcribed, and emerging themes and subthemes were continually revised and reviewed as further interviews were conducted and subsequently analyzed. Research notes made during interviews allowed for further refining of themes.

**Figure 1.**
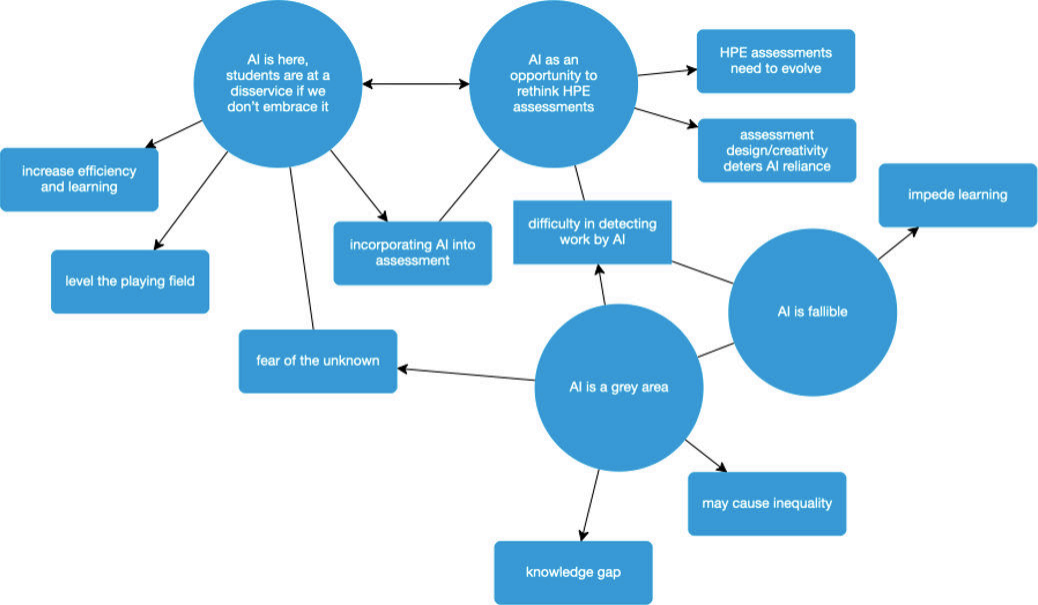
Thematic map. AI: artificial intelligence; HPE: health professions education.

In phase 5, the datasets were reviewed, analyzed, and visualized on a thematic map (Figure 1), enabling further refinement of coherent themes and subthemes without repetition. Phase 6 involved writing up the analysis. Reflexively, CPs’ lived experience straddling educator and learner roles while undertaking HPE assessments in an evolving AI era may have helped co-construct knowledge and understanding and enabled development and depth to the codes and themes.

### Ethical Considerations

Data were stored securely on a university password-protected platform. Ethics approval was granted by the University College London Research Ethics Committee (ethics 23511/011). Participation was voluntary and without compensation, and informed consent was obtained from all participants. Consent for publication was obtained from all participants; no identifiable data were used.

## Results

### Overview

Eight semistructured interviews were conducted via Microsoft Teams between January and March 2024 (4 educators [E] and 4 learners [L]), lasting 30 to 60 minutes (Table 1). Six doctors and 2 nurses across 6 different higher institutes within the United Kingdom were interviewed. Data saturation was reached after 8 interviews, reaching informational redundancy. The participants had a range of prior AI knowledge; however, the distinction between learners and educators was blurred, as all educators had previously been learners in HPE, and all learners were currently educators in some capacity, which may have led to more analogous perspectives. The dual role of interviewee as both educators and learners may, therefore, have led to theme convergence.

The iterative process of data analysis reduced 15 subthemes to 10 and 6 themes to 4. The four themes are (1) AI as an opportunity to rethink HPE assessments; (2) AI is here, students are at a disservice if we do not embrace it; (3) AI is a gray area; and (4) AI is fallible.

As seen in the thematic map (Figure 1), some themes and subthemes have strong associations (bidirectional arrows) and affiliations (straight lines), demonstrating the interconnection between concepts.

**Table 1.** Participant demographics.

Participant	Length of interview (mean 35.5 min, SD 10.16)	Clinical background
L1	32 min	Nurse
L2	41 min	Doctor
L3	57 min	Doctor
L4	24 min	Doctor
E1	25 min	Doctor
E2	33 min	Doctor
E3	30 min	Nurse
E4	42 min	Doctor

#### Theme 1: AI as an Opportunity to Rethink HPE Assessments

##### Overview

All participants, except 1, had only written assessments in their HPE course (7/8, 88%): predominantly essays, with some research proposals and literature reviews. AI represented an overdue opportunity to “rethink and go back to the drawing board about why we’re assessing and what people are learning and how” (L2). HPE assessments have remained static, and all participants apart from one felt that the established method of predominantly essay-based assessments was not the most appropriate way to assess educators for the modern world, which included rising AI use and difficulty detecting its use, exemplified by L3: “Are they [assessments] valid or are we simply just gonna get a whole load of ChatGPT generated work?”

The desire for internal change existed within HPE prior to recent external pressure of AI, and all participants (8/8, 100%) wanted HPE assessments that constructively aligned with the development of critical-thinking educators, proficient in pedagogy, especially in practical application.

There were 2 subthemes: HPE assessments need to evolve and assessment design or creativity drives AI use.

##### Subtheme 1.1: HPE Assessments Need to Evolve

There was a consensus that assessments in HPE should evolve from being predominantly essay-based for a multitude of reasons: written assessments were easier to produce using AI, disadvantaged learners who struggled with writing were often assessed on writing ability as opposed to content, lacked creativity, and might not demonstrate knowledge or reflect engagement in the course.

All participants except 1 (88%) felt it was important to retain some written assessments, such as essay writing, to hone critical and academic writing skills and prepare learners for future academic pursuits while also acknowledging the educational impact written assessments had in driving their learning. Keeping assessments as they are is perceived as the easier option, with less resistance and easier to mark than potential alternatives: “[T]here’s no criticism of people that are just continuing and not...putting their heads above the parapet because at the end of the day it is a complex task” (L2)

##### Subtheme 1.2: Assessment Design or Creativity Deters AI Reliance

Lack of diversity in assessment types combined with lack of time may drive learners to turn to AI, described by L1: “When you go in and you go ‘Oh no, another 4000 word essay’...you're just like, ‘Oh my...I don't wanna do this’ and therefore you're more likely to turn to...generative AI.”

Careful assessment design and creativity to incorporate AI “so that it doesn’t diminish learning and assessment” (L1) will also create assessments that are less amenable to being produced purely by AI, without having to ban AI use outright.

Suggestions were made for assessments to better reflect real life (Textbox 1); within research, this may be writing abstracts, posters, or journal articles, and within clinical teaching, collaboration and group work. However, these “might be challenging for [educators] because it would be a complete fundamental change” (L2). Lack of creativity in current assessments may drive AI use, and further steps could be taken to demonstrate student input, such as in presentations or explaining how they interacted with AI. Some educators described how they were reviewing their assessments in light of AI, but most were currently only at the discussion stage.

Textbox 1. **Suggested alternative health professions education assessment types.**

The following are the health professions education assessment types:

- Viva (oral examination)
- Poster presentation
- Video
- Collaborative working
- Oral presentation
- Teaching observation and feedback
- Project work or group research
- Abstract or journal article

### Theme 2: AI Is Here, Students Are at a Disservice If We Do Not Embrace It

#### Overview

AI is part of everyday life, both educationally and clinically. No participants described their current HPE course as either incorporating or teaching AI use, with concern by some participants that educators may not acknowledge AI, allowing courses to continue as they are, perhaps driven partly by fear. Participants felt that if AI is not incorporated into teaching and, therefore, assessment, learners would be placed at a disadvantage, and the HPE course may be subsequently less relevant and attractive to future learners.

*I don’t think it’s right to say, don’t use generative AI at all, because I just don’t think that’s realistic.*

[E4]

There were 3 subthemes: incorporating AI into assessment, increasing efficiency, and learning and leveling the playing field.

#### Subtheme 2.1: Incorporating AI Into Assessment

A total of 2 (50%) learners had used AI within their HPE course for learning but not summative assessment; 1 did not use it in their assessment, as they felt it was unnecessary. Of all, 2 (50%) educators described using it in their teaching and 1 using it to aid assessment design. Participants discussed how AI could be incorporated into the assessment process in Textbox 2.

Textbox 2. **Suggested uses by participants of artificial intelligence in health professions education assessments.**

The following are the suggested uses by participants of artificial intelligence (AI) in health professions education assessments:

- AI to assist in image creation or graphical depiction of research
- AI to create a poster to present work or research
- Integration of AI within assessment—marks allocated to AI use or combine AI input with human oversight or feedback
- Demonstrate critical analysis of AI output (ie, demonstrate reworking of prompts and rationale; editing, fact-checking, or reflection on AI output)
- Use AI as an exemplar assessment to critique (may be an example of “bare minimum pass”)
- AI to assist educators to mark assessments and provide learner feedback
- Provide learners with personalized feedback on draft assessment

#### Subtheme 2.2: Increase Efficiency and Learning

Participants suggested how AI can increase efficiency in assessments for both educators and learners (Textbox 3). The educational impact of incorporating AI into the assessment process was described by L1: “(AI) would sort of relieve the pressure of time, so then you can do those more exciting, interesting things...and in turn makes students more motivated.”

A total of 6 (75%) participants had used AI in some capacity within HPE. Textbox 4 outlines how they used AI in HPE assessments to facilitate learning, highlighting similarities between educators and learners.

Participants discussed how HPE courses aimed to develop competent health professions educators to teach health professions with the ultimate aim of improving patient care.

Participants also highlighted the need to retain human educator oversight and involvement within HPE and assessment, to avoid losing the “human touch,” as AI alone would be too impersonal, decreasing motivation and learning. As described by E4: “I don’t think [AI] would get rid of jobs completely.”

Table 2 outlines participant suggestions on how AI could be used alongside the learner or educator to facilitate assessment *as*, *for,* and *of learning*, as similarly seen in the literature [25].

Textbox 3. **Suggestions on how artificial intelligence may increase efficiency.**

The following are the suggestions on how artificial intelligence may increase efficiency:

- Conduct literature search, identify papers
- Project outlines
- Access information, compile information
- Summarize concepts or ideas
- Write first draft (eg, introduction, reference)
- Develop presentations, poster, or slides
- Mark assessments
- Provide evaluation or feedback

Textbox 4. **How AI is being used in health professions education assessments.**

The following illustrates the ways in which artificial intelligence (AI) is being used in health professions education assessments:

- Offer new perspectives and ideas
- As an alternative to search engines
- To test and check hypotheses
- To confirm and check current knowledge
- Generate an answer to a question
- To provide a critical analysis
- To generate initial ideas
- Rephrasing, editing, finessing
- Prompt and creative outlet in assessment creation

**Table 2.** Suggested uses of AI^a^.

AI use	Practical suggestions
AI as a coteacher	To clarify or explain concepts that are unclear
AI as a tutor or mentor	To review work and provide suggestions
AI to encourage self-directed learning	To provide guidance that can enable self-directed learning (assessment as learning)
AI as an assistant	To produce an initial draft or outline that can be refined or edited
AI as an aid (for both educator and learner)	Assessments may be produced by AI then refined by learner, or be the “first marker” for assessors

a AI: artificial intelligence.

#### Subtheme 2.3: Level the Playing Field

There are opportunities for AI to help level the playing field, such as for students with English as a second language, as also seen in other literature [26]. It may also “offer a bit more objectivity [and] consistency” (E4) and remove any conscious or unconscious bias from assessors if used in marking. This was particularly relevant as participants felt that marking of essays in HPE lacked objectivity and reliability.

### Theme 3: AI Is a “Gray Area”

#### Overview

All participants (8/8, 100%) felt that AI use and application within HPE assessments was a “gray area” and felt a lack of “formal guidance” (E2) from an institutional level. Half the educators (2/4, 50%) and 3 (3/4, 75%) learners knew their institution’s stance on AI use within assessment, but many still felt uncertain about how to tangibly translate it to practice. E1 states: “no one quite knows what we should and shouldn't be doing.” Learners lacked confidence and wanted guidance from educators to ensure they did not get accused of academic misconduct, while educators found themselves relying on learners for guidance or education on AI too. The lack of clarity and confusion on AI use in assessment was identified as a loophole, meaning that learners could potentially use AI with a “get-out clause,” resulting in inequality if some students are using it and others are not.

There were concerns about whether AI use constituted plagiarism, due to a lack of familiarity and clarity regarding AI application and authorship. This created a sense of unease among most participants, making educators and learners reluctant to use it, even if permitted, for fear of incidental academic misconduct. Clear direction and boundaries on AI use had to come from an institutional level, in collaboration with subject matter experts and educators, but participants were concerned that “getting stakeholder buy-in would be quite a hard thing” (E4).

There were 4 subthemes: difficulty in detecting work by AI, knowledge gap, fear of the unknown, and may cause inequality.

#### Subtheme 3.1: Difficulty in Detecting Work by AI

There was concern from both learners and educators about the difficulty of detecting AI in HPE assessments, with participants recommending the implementation of AI detection tools. However, participants were aware that AI detectors were often not used or were fallible. Some learners had to sign a statement of originality, while some universities allowed the use of AI with appropriate referencing. However, there was confusion among participants about “how to use [AI] effectively” (E4); “if they [students] are even allowed to use it or is it a type of plagiarism?” (E4); and if referenced, if that would be “good enough” (L4).

Participants noticed a formulaic “hallmark” style to AI output. Yet educators did not feel confident that they would be able to identify whether AI had been used in assessments, suggesting they would rely on knowing the student’s voice or identifying phantom references to identify AI use.

#### Subtheme 3.2: Knowledge Gap

The knowledge gap was a cause for concern among all participants, with both the newness of AI and fear of the unknown driving a wedge between those who have embraced it and those who have not. No learners described AI being formally taught on their HPE course, and those perceived as knowledgeable or familiar with AI were deemed more technologically savvy and self-taught. Most participants had not used AI as they felt “underconfident” (L4), and the more AI-confident participants displayed an innate interest in discovering AI’s capabilities.

There was concern from some educators about the knowledge gap in AI, both personally and among their students. Only 1 (25%) educator had discussed AI with HPE colleagues and reported their colleagues were fearful and uncertain of its use, so knowledge was not being shared or cocreated among communities. Educators thought that learners, especially those who were younger, may have more knowledge on AI and that faculty “need to keep up” (E4) and “upskill” (E1).

There was mixed perception regarding whether learners or educators in HPE were more AI literate, with a likely range of knowledge within both cohorts. In this study, neither group demonstrated superior AI knowledge. Positively, both learners and educators were keen for teaching on AI at an institutional level, especially as educators felt that the students were expecting them to be up to date and teaching them. Participants wanted guidance on AI use: learners from educators and educators from their institutions—in the form of policy and AI literacy workshops.

#### Subtheme 3.3: Fear of the Unknown

AI’s newness created fear and uncertainty for both educators and learners in both how and when to use it, and the inability to keep up with its constant evolution. Due to a lack of knowledge, they felt scared not only of how to use it within HPE assessment but also of getting in “trouble” for using it.

#### Subtheme 3.4: May Cause Inequality

Learners with more knowledge or access to AI may have an advantage within HPE assessment and “create a bit of an unfair playing ground” (E2). Rather than leveling opportunities, AI may further drive inequality within HPE assessments because those who can afford more advanced AI platforms or have higher AI literacy levels may be better able to use it more successfully and evade detection if required.

### Theme 4: AI Is Fallible

#### Overview

Participants discussed the merits and drawbacks of AI, specifically the inability to trust the output “because it’s never perfect” (L1), often requiring a human to fact-check and review the output, as “50% of it is excellent, 50% of it is rubbish” (E1).

There was 1 subtheme: impede learning.

Participants were very cautious of AI and felt unable to trust its output, partly due to “hallucinations.” Participants highlighted concerns that some learners may not be aware they must critically appraise AI output, especially if in a rush or under time pressure, which would further learner inequality and “gray areas” based on AI literacy.

#### Subtheme 4.1: Impede Learning

There was concern about the educational impact of assessments, as AI use within HPE assessment may not demonstrate learning. If learners use AI to “generate their assignment...are they going to learn anything?” (E3) and potentially pass an HPE course with minimal input or knowledge. HPE degrees should only be awarded to those “deserving of that degree...(who) display a certain level of original ability (E2).” However, some participants felt that AI cannot demonstrate high-level thinking or criticality, so the threat was lessened.

Participants highlighted the irony that educators may be using AI to mark AI-generated assessments; hence, learners may be able to pass an HPE course with minimal commitment or learning if only written assessments are required.

## Discussion

### Principal Findings

#### Implications for Assessment Design

AI highlights the internal need for a radical overhaul of HPE more broadly, with the assessments reflecting as such. AI, alongside ever-increasing medical knowledge, represents the need to transition from memorizing information to knowledge utilization [27] and has provided the opportunity to reconsider HPE assessments, while incorporating AI, to upskill learners. Reconsidering HPE assessment design in light of the more recent external pressure of AI offers a rethink of both *how* and *what* we are assessing to ensure the course is suited to develop critical-thinking, better-prepared educators.

The findings build on longstanding concerns that “traditional” assessment methods (eg, essays) within master’s programs lack consistency and that alternative assessment methods are preferable to improve both motivation and fairness [28]. However, as participants wanted to retain some written assessments to develop critical analysis through essay writing, perhaps we can use AI’s output to develop those skills [8]. Education does students a disservice if new technologies, such as AI, are not embraced. Creating authentic, student-centered assessments will not only cultivate critical thinking, encourage learning, and decrease academic misconduct but also better prepare learners for future practice if they reflect genuine tasks undertaken by the HPE community [29,30]. AI also influences clinical practice, improving diagnostic efficiency and accuracy [15], so it is disingenuous not to use it in educating future educators.

Incorporating AI into assessment provides an opportunity to develop creative assessments that appeal to students’ different strengths and increase engagement while decreasing overreliance on AI to write the assessment. Interestingly, participants felt that current GenAI programs would not be able to write an entire essay sufficiently critical to successfully pass an HPE assessment. However, using targeted follow-up prompts, ChatGPT has been shown to produce a reasonable academic piece of work within 2 to 3 hours [31], and despite some inaccuracies, this is much quicker and with minimal expertise or effort than a human-written essay.

AI has inevitably influenced HPE assessments, forcing educators and learners to decide whether to embrace it or ignore reality. The findings corroborate current literature [32] in demonstrating how most participants were already embracing AI, suggesting ways it can be incorporated into assessment creation, design, implementation, and marking, with an understanding that in the future, AI and assessment will be inextricably linked. AI use, especially for repetitive or onerous tasks, may decrease the “cognitive-load” and create time and space for more creativity within assessments. Such “cognitive offloading” is described by Risko and Gilbert [33] as the process whereby a physical action (such as using an LLM) can “reduce the cognitive demands of a task” [33].

The intended learning outcomes of the HPE courses should be reconsidered to develop educators who will thrive in the era of learning with and from AI and, therefore, incorporate AI in assessments to constructively align with such teaching and learning [8]. However, overreliance on AI may be detrimental to fundamental learning [30], so educators need to carefully consider how AI applications best align with the intended learning outcomes.

Findings that the “personal touch” from educators was strongly valued by learners reflect similar research, which showed how online learning, specifically online assessments, that overrely on technology and lack human presence are “dehumanized” and detrimental to student learning [34]. Human connection has been demonstrated as the most important factor in engagement and student learning [35], and the collaboration between educators and AI in assessment design and delivery may not only promote community belonging for learners but also enhance engagement in both assessment and learning [34]. This supports findings that AI and educators must coexist to facilitate learning.

#### Institutional Policy Recommendations

The uncertainty surrounding AI use and plagiarism reflects current thinking that AI use requires a full reconsideration of what plagiarism now represents [26]. Perkins [26] suggests that clear acknowledgment of AI use by learners should not be considered academic misconduct by institutions, instead emphasizing that learning outcomes may not be met if AI generates such assessments. AI hesitancy in this study may reflect that health care professionals must hold honesty and integrity central to their vocation, and academic misconduct can be referred to their governing body (for doctors, the General Medical Council), inciting fear of accidental misuse. AI will likely always be one step ahead, and the deficiencies in AI detection tools make detection like a game of cat and mouse (described by [36]). Furthermore, relying on educators detecting AI via methods such as identifying hallucinated references is highly subjective due to educators’ differentials and may exacerbate marking inequalities. Efforts should be redirected into collaborating and incorporating AI into assessment design and content, encouraging assessment as and for learning.

Both educators and learners wanted to increase their AI literacy and recognized an opportunity for increased learning, reflecting wider literature [32], but acknowledged the complexity of this task and potential resistance to change at an institutional level. AI inequality in learners can be addressed by university access and increased AI literacy. Participants wanted the direction to come from their institutions; however, a study showed that in times of change (to online assessment), new assessors sought support and guidance from more informal networks within the communities of practice (eg, colleagues in similar situations) as opposed to formal networks (eg, university experts) [37]. Therefore, creating assessor and learner CoPs to discuss and share AI practice could develop understanding and confidence with AI, with such support systems in new online learning environments helping to foster connection and engagement [35].

#### Implication for Educator Training

There are multiple resources from the online community on how to use GenAI (such as ChatGPT) within HPE; however, these assume a certain level of digital literacy of its readers (ie, [38]). An informal assessor CoP, such as between colleagues, is an easy and effective starting point to discuss and share practice, developing AI literacy and confidence that all participants desired, and such communities have demonstrated success in facilitating change within HPE teaching practices [39].

AI is a phenomenon that the assessor community must respond to, with institutional guidance. In a CoP, learners usually learn from the “masters” in the community; however, this may be flipped for AI, as it seems to be the masters dragging their feet. A CoP can facilitate successful institutional-level change in HPE, though this requires institutional appetite for change and keen faculty who are supported with time and resources to reflect on experiential learning [40]. Current literature describes approaches to developing faculty AI literacy, improving educator confidence and ability to integrate AI into the curricula [41], alongside the importance of collaboration and coproduction of knowledge between stakeholders (including AI specialists) to ensure current and evolving AI literacy and application [42].

Early AI adopters may need to drive such an assessor CoP to develop a critical mass for AI to become widely accepted and to establish acceptable uses for learning. The assessor community needs to work more widely with colleagues who are at the forefront of or more comfortable with AI to drive change. This will represent interdepartmental, or even inter-university, cross-collaboration [43].

### Actionable Strategies in HPE to Guide Assessment Redesign

The following are the actionable strategies in HPE to guide assessment redesign:

- “Humanizing” AI assessments involves integrating AI into assessment format and content, while ensuring the human component remains. This would likely improve both educator and learner experience, while deterring from full reliance on AI use.
- Institutions should hold AI literacy workshops to increase AI literacy and access, as workshops and teaching for both educators and learners will further drive collaboration and successful CoPs.
- Raise educator awareness and understanding of the ethical issues around AI and how these can impact learner acceptance of AI. There is a range of ethical issues, such as environmental cost, bias, data protection, digital inequalities, etc, that many educators are either unaware of or feel are the institutional decisions to be made. Learners have their own thoughts on these ethical issues, which impact their willingness and acceptance of AI use.
- Establish or revisit assessor CoPs (driven by early AI adopters) to encourage sharing of AI knowledge, which will likely require institutional buy-in. Such CoPs may challenge the status quo, with “masters” learning from “novices,” and early AI adopters working collaboratively with assessment experts to improve and produce authentic HPE assessments.
- Retain the essay as one of a multitude of assessments, with careful essay design to use AI for “cognitive offloading” (for educators and assessors) and enable higher-order critical thinking. AI has the potential to be an effective tool within learning and assessment, and some approaches can help improve AI’s output: improving prompts, critically reviewing output for accuracy, and checking references.

### Future Research Needs

The following are the future research needs:

- Consider how GenAI is influencing postgraduate HPE assessments outside the United Kingdom
- Consider how GenAI is influencing HPE assessments with perspectives from educators and learners across different health professions

### Limitations

There was potential educator and learner response bias among those who felt more confident discussing AI. The research was specific to postgraduate HPE, so the opinions of undergraduates and students from different courses may differ. Interviews were conducted by a single interviewer (CP) as part of her master's thesis, and despite efforts at reflexivity, there may have been interviewer bias. The study is based on 8 interviews from institutions across the United Kingdom, predominantly doctors, so generalizability to different countries and courses may be limited. Furthermore, it is difficult to extrapolate group comparison due to the role duality of learner-educators, as well as the small sample size of only 4 participants per group. While it was not possible to obtain the assessment format from all HPE courses, as participants were from different HPE courses, they were able to provide an overview of various formats.

### Conclusion

Key findings indicate that educator and learner perspectives agree on the opportunities and challenges presented by GenAI: AI is here, students are at a disservice if we do not embrace it, AI as an opportunity to rethink HPE assessments, AI is a gray area, and AI is fallible. Participants consider AI to offer the opportunity for an overdue reevaluation of predominantly written-based HPE assessments to better reflect real clinical and academic life and develop educators who are going to enter the modern workforce in which AI is ever present. Developing an assessor CoP will allow for shared AI practice and knowledge cocreation, building both educators’ and learners’ confidence in this new field.

## Supplementary material

10.2196/87121 Multimedia Appendix 1 Interview schedule.

10.2196/87121 Checklist 1 COREQ checklist.

## Abbreviations

AI: artificial intelligence
CoP: communities of practice
COREQ: Consolidated Criteria for Reporting Qualitative Research
GenAI: generative artificial intelligence
HPE: health professions education
LLM: large language model
