# Supplementary material for: Opportunities and Challenges of Generative AI in Postgraduate Health Professions Education Assessments From Educator and Learner Perspectives: Qualitative Study
Source: JMIR Form Res. 2026 May 6;10:e87121. doi: 10.2196/87121 (PMC13148761; doi:10.2196/87121)
Supplement: Multimedia Appendix 1 [file formative-v10-e87121-s001.docx]

**Appendix: Final Interview schedule**

**Semi-structured Interview schedule**

**Recruitment, pre-interview preparation**

- Aim to recruit 3-4 participants from each group (learner and educator) to attend semi-structured interviews
- Send a Project Information Sheet to all participants who are interested. Then send a consent form to those who wish to attend an interview via email containing a link to the consent form on REDCap which must be signed prior to the interview.
- There will be a mutually agreed time to conduct the interview
- Email interview invites to participants with MSTeams link for the virtual interview

**Set up:**

- The interview will be conducted virtually on MSTeams
- CP, as the only researcher, will be the interviewer
- The interview will last approximately 30-40 minutes

**Interview:**

- **Introduction: “**Welcome to the interview and thank you for participating. My role as interviewer is to ask questions, allow space for your response and explore your perspectives. The interview is semi-structured which means I have a list of overall questions to ask but allows the freedom for the conversation to flow and develop naturally, depending on your answers and we don’t have to follow the order rigidly. I am here in an impartial role and there are no right or wrong answers, so please feel free to speak freely.”
- **Research question and goals:** “What are educator and learner perspectives on the opportunities and challenges presented by generative AI in postgraduate Health Professions Education assessments?”
- “Health Professions Education here is a postgraduate course for healthcare professionals at higher education institutes which explores teaching and learning theory and practice, and passing required assessments results in a recognized teaching qualification”
- “Generative AI here relates to algorithms that generate new output/content, some well-known examples include ChatGPT and Bard.”
- “Your input today will help contribute towards the understanding of how generative AI may influence current and future HPE assessments, and what this means for the future of HPE assessments.”
- **PIS/consent form**: “Did you have any questions about the PIS or consent form? The conversation will be audio-video recorded by MSTeams and automatically transcribed. I will pseudonymized and edit the transcript, and then analyse the data for themes. Please switch your camera off so that the video recording will just be of the screen with your display name on it.
- The audio-video recording will be deleted after it has been transcribed. You may ask to withdraw your data until two weeks after your interview, after which data analysis will have started. Please refer to the PIS for further information.”
- “I would like to remind you that participation is entirely voluntary. You may stop at any point without reason, and either take a break and re-join, or end the interview. I can signpost you to support for any concerns raised today, please let me know or email me at XXXXX”
- **Confidentiality** “Please remember that the interview is confidential. Assurances on confidentiality will be adhered to unless evidence of wrong-doing or harm is identified. If this happened, as researcher with a duty of care, I will then be obliged to contact the relevant bodies. I will inform you of any decision that might limit your confidentiality.”
- **“Any final questions or concerns before we begin? Please switch off your camera if you haven’t already done so”**

**Final Questions**

**Current knowledge, expectations, concerns/worries/reservations**

Q1) Could you let me know your current role on your HPE course?

- Are there a mix of HPEs?
- Please may you give me a general overview of the current assessments on your HPE course?
- Do you think these are effective assessment methods? Why/why not? Does this change from a learner or educator point of view?

Q2) Are you familiar with the concept/practicalities of generative AI?

- Do you think your educators or students are familiar with AI? Are your colleagues familiar with it? What are your institutions saying?

Q3) Have you used generative AI in your current role as educator/learner in the health professions education (HPE) course?

- If yes – in what capacity has it been used?
- If not – why not?

Q4) Have you used generative AI in relation to an assessment in your current role as educator/learner in the health professions education course?

- If yes – in what capacity has it been used?
- If not – why not?

Q5. Are you aware of how generative AI is being used by your colleagues within health professions education assessments?

Q6. What do you think are the challenges of generative AI for educators in HPE assessments?

Q7. What do you think are the opportunities of generative AI for educators in HPE assessments?

Q8. What do you think are the challenges of generative AI for learners in HPE assessments?

Q9. What do you think are the opportunities of generative AI for learners in HPE assessments?

Q10. How do you think generative AI may influence the future of HPE assessments?

Q11. Do you think we need to change how we assess in HPE?

Q12. Do you think we can continue to assess via essay-based assignments (ie literature reviews, essays etc)?

- If yes – why
- If not – why not?

Q13. Do you have any suggestions of how we could change HPE assessments considering generative AI?

Q14. Any other thoughts or comments regarding what we have discussed above?

Q15. Any other thoughts or comments not discussed above regarding generative AI and HPE assessments?

**Closing the interview**

- **“Thank you for participating. That is the end of the interview, I am now going to stop recording.”**
